# THE POSSIBILITY OF THE EVOLUTION OF NEUROLEPTIC MALIGNANT SYNDROME DURING THE CONCOMITANT USE OF CLOZAPINE WITH LITHIUM SALTS

**DOI:** 10.1192/j.eurpsy.2023.2131

**Published:** 2023-07-19

**Authors:** A. Tomcuk, J. Đedović, A. Macic

**Affiliations:** Special Psychiatric Hospital Kotor, Kotor, Montenegro

## Abstract

**Introduction:**

The neuroleptic malignant syndrome is a rare but potentially the most dangerous complication of neuroleptic use. The first descriptions of this disorder were given by Delay and colleagues in the 1960s, calling it “hypertonic akinetic syndrome” and characterizing it with hyperthermia, extrapyramidal symptoms, altered mental status, and autonomic dysfunction. Current knowledge unequivocally shows that NMS most often occurs with the use of first-generation antipsychotics, especially in parenteral form. After the appearance of this disorder, the usual practice is to transfer the patient to monotherapy with clozapine, excluding antipsychotics from the chemical group that led to NMS.

However, there are isolated reports of the occurrence of this syndrome in patients on concomitant therapy of clozapine with lithium salts.

**Objectives:**

The main objective is to present possible case of NMS in patients with concomitant use of clozapine and lithium

**Methods:**

Case report: M. K., born in 1984, graduated from high school, unmarried, without permanent employment. In 2003, after the parenteral application of Depo preparation, he developed NMS. He was hospitalized at the Institute of Psychiatry in Belgrade when he was diagnosed with psychosis of the schizophrenic cycle. After discharge from the hospital, stable clinical remission persists with maintenance therapy with clozapine. In July 2018, after a short stay abroad, he developed a pronounced behavioral disorganization and was forced to be admitted to our institution.

On admission and in the initial phase of hospital treatment, the clinical picture is dominated by intense psychomotor restlessness, acoustic hallucinatory experiences, dysphoric-euphoric mood, and uncontrolled verbal and physical aggression towards medical staff and other patients. He was treated with clozapine, which was gradually titrated up to 600 mg daily, with benzodiazepine support (lorazepam 10 mg p.d.) without significant improvement, medical sedation, and lithium salts were introduced into the therapeutic protocol.

**Results:**

Immediately afterward, psychomotor inhibition occurs, accompanied by somnolence, mild hypotension, sinus tachycardia, and subfebrile temperatures, without signs of muscle stiffness. Lab. analysis of CpK 628, other findings within reference values. Excluded psychopharmaceuticals from the therapeutic protocol, introduced detox. procedures and III generation cephalosporins, after which the patient’s condition stabilizes.

**Conclusions:**

It is important to bare in mind the possibility of NMS in patients with concomitant use of clozapine and lithium

**Disclosure of Interest:**

None Declared

